# Differential Function of Endogenous and Exogenous Abscisic Acid during Bacterial Pattern-Induced Production of Reactive Oxygen Species in *Arabidopsis*

**DOI:** 10.3390/ijms20102544

**Published:** 2019-05-23

**Authors:** Leitao Tan, Qiuping Liu, Yufeng Song, Guangzhen Zhou, Linli Luan, Qingbei Weng, Chaozu He

**Affiliations:** 1 Hainan Key Laboratory of Sustainable Utilization of Tropical Bioresources, Hainan University, Haikou 570228, China; songyufengqqq@163.com (Y.S.); guangzhenvip@163.com (G.Z.); ll0527ll@163.com (L.L.); 2 Institute of Tropical Agriculture and Forestry, Hainan University, Haikou 570228, China; 3 School of Life Science, Guizhou Normal University, Guiyang 550025, China; liuqiuping0999@163.com (Q.L.); wengqb@126.com (Q.W.)

**Keywords:** abscisic acid, reactive oxygen species, *Arabidopsis*, *Pseudomonas syringae*, callose deposition, salicylic acid

## Abstract

Abscisic acid (ABA) plays important roles in positively or negatively regulating plant disease resistance to pathogens. Here, we reassess the role of endogenous and exogenous ABA by using: 35S::*ABA2*, a previously reported transgenic *Arabidopsis* line with increased endogenous ABA levels; *aba2-1*, a previously reported *ABA2* mutant with reduced endogenous ABA levels; and exogenous application of ABA. We found that bacterial susceptibility promoted by exogenous ABA was suppressed in 35S::*ABA2* plants. The 35S::*ABA2* and *aba2-1* plants displayed elevated and reduced levels, respectively, of bacterial flagellin peptide (flg22)-induced H_2_O_2_. Surprisingly, ABA pre-treatment reduced flg22-induced H_2_O_2_ generation. Exogenous, but not endogenous ABA, increased catalase activity. Loss of nicotinamide adenine dinucleotide phosphate oxidase genes, *RBOHD* and *RBOHF*, restored exogenous ABA-promoted bacterial susceptibility of 35S::*ABA2* transgenic plants. In addition, endogenous and exogenous ABA had similar effects on callose deposition and salicylic acid (SA) signaling. These results reveal an underlying difference between endogenous and exogenous ABA in regulating plant defense responses. Given that some plant pathogens are able to synthesize ABA and affect endogenous ABA levels in plants, our results highlight the importance of reactive oxygen species in the dual function of ABA during plant-pathogen interactions.

## 1. Introduction

Abscisic acid (ABA) is a class of metabolites known as isoprenoids that controls seed maturation and germination, as well as adaptation to abiotic stress [1,2]. As a stress-related hormone, ABA plays a negative or a positive role in plant disease resistance, depending on pathogen lifestyles. The ABA is formed by cleavage of C_40_ carotenoids in the 2-C-methyl-d-erythritol-4-phosphate pathway [3,4]. Zeaxanthin is produced as a trans-isomer via β-carotene. Zeaxanthin epoxidase (ZEP) catalyzes the conversion of zeaxanthin and antheraxanthin to violaxanthin in plastids [5,6]. The formation of cis-isomers of violaxanthin and neoxanthin may require a neoxanthin synthase and an isomerase [2]. The cis-isomers of violaxanthin and neoxanthin are oxidized by nine-cis-epoxycarotenoid dioxygenase (NCED) to generate a C_15_ product, xanthoxin, and a C_25_ metabolite [7]. The Xanthoxin is further converted by a short-chain alcohol dehydrogenase/reductase 1 to abscisic aldehyde [8,9]. The final conversion of abscisic aldehyde to ABA is catalyzed by abscisic aldehyde oxidase (AAO) and a molybdenum co-factor sulfurase is required to ensure AAO activity [10,11,12,13].

ABA biosynthetic mutants have been widely used to investigate the role of ABA in plant defense responses [14,15,16]. Following *Leptosphaeria maculans* infection, the *Arabidopsis ZEP* mutant *aba1-3* showed enhanced susceptibility, impaired callose deposition, enhanced expression of the SA-dependent pathogenesis-related protein 1 gene *PR1*, and reduced expression of the jasmonic acid and ethylene (JA/ET)-responsive defensin gene *PDF1.2* [17]. Compared with wild-type *Arabidopsis*, many defense-related genes regulated by SA, JA, and ET were constitutively up-regulated in *aba1-6* [18]. Compared with wild-type tomato, *PR1a* expression in the *AAO* gene mutant *sitiens* was induced with a very low concentration of the SA-analog benzo(1,2,3)thiadiazole-7-carbothioic acid *S*-methyl ester (BTH) [19]. Compared with wild-type *Arabidopsis*, JA accumulation induced by the virulent *Pseudomonas syringae* pv. *tomato* (*Pst*) strain DC3000 was lower in the molybdenum co-factor sulfurase *ABA3* gene mutant *aba3-1* [20]. Recently, it has been reported that *aba3-1* exhibited enhanced expression of SA-responsive genes when challenged with *Golovinomyces cichoracearum* [21]. Nevertheless, it must be noted that ABA deficiency often leads to growth or developmental defects [16], as seen with *aba2-2* exhibiting a wilting phenotype [22]. Developmental differences between mutant and wild type might result in a biased evaluation of ABA function.

An *ABA2* transgenic *Arabidopsis* line 35S::*ABA2* has been demonstrated to enhance endogenous ABA levels and grow normally [23], compared to wild-type plants. In this study, the transgenic line 35S::*ABA2*, the *ABA2* gene mutant *aba2-1* exhibiting a dwarf and wilting phenotype [10], and exogenous application of ABA, were together used for reassessment of endogenous and exogenous ABA in plant defense responses.

## 2. Results

### 2.1. Endogenous ABA Antagonizes Exogenous ABA-Aggravated Susceptibility

**Figure 1:**
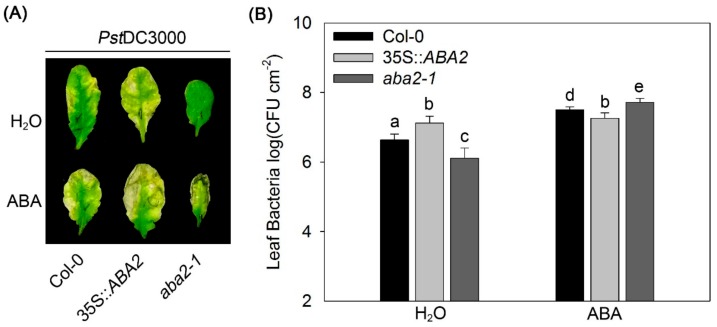
Effects of endogenous and exogenous abscisic acid (ABA) on *Pst*DC3000 resistance in *Arabidopsis* at 3 dpi. (**A**) Disease symptoms and (**B**) bacterial growth. Wild-type (Col-0), the *ABA2* transgenic line 35S::*ABA2*, and the *ABA2* gene mutant *aba2-1* were treated with distilled water (H_2_O) or ABA for 4 h prior to inoculation with *Pst*DC3000. Error bars indicate the standard error, and values were based on at least eight independent replicates. Different letters indicate values that are significantly different (*p* < 0.05) from each other as determined by one-way ANOVA. These experiments were performed at least three times.

ABA negatively regulates resistance to biotrophic bacteria *P. syringae* in *Arabidopsis* and exogenous application of ABA restores disease susceptibility of ABA-deficient mutants [14,15,16]. To better understand the role of ABA in plant disease resistance, wild-type (Col-0), 35S::*ABA2*, and *aba2-1 Arabidopsis* plants were pre-treated with distilled water (as control) and 50 µM ABA, prior to inoculation with virulent *Pst*DC3000. Pathogenicity analysis showed that in the water controls, 35S::*ABA2* plants displayed severe chlorosis and enhanced bacterial growth, whereas *aba2-1* had no observed symptoms and had lower bacterial numbers, compared to wild-type (Figure 1). Following exogenous ABA pre-treatment, *aba2-1* exhibited severe necrosis and strongly enhanced bacterial numbers compared to wild-type (Figure 1). Meanwhile, enhanced disease symptoms, but not bacterial numbers, were exhibited in 35S::*ABA2* under ABA treatment (Figure 1). These results indicate that endogenous and exogenous ABA overlap with each other in repressing bacterial resistance, but it is also possible that endogenous ABA antagonizes exogenous ABA-promoted susceptibility to *Pst*DC3000, at least partly, in *Arabidopsis*.

### 2.2. Endogenous ABA Enhances, but Exogenous ABA Represses Bacterial Pathogen-Associated Molecular Pattern (PAMP)-Induced ROS Burst

**Figure 2:**
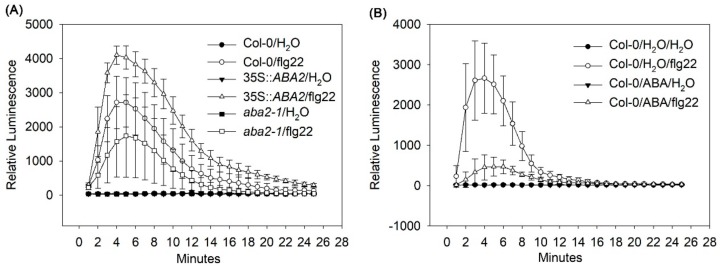
Effects of endogenous and exogenous abscisic acid (ABA) on H_2_O_2_ production induced by flg22 in *Arabidopsis*. (**A**) Endogenous ABA and (**B**) exogenous ABA. For analyzing exogenous ABA, wild-type (Col-0) plants were treated with distilled water (H_2_O) or ABA before detecting flg22-induced H_2_O_2_ production. Error bars indicate the standard error, and values were based on at least eight independent replicates. These experiments were performed at least three times.

ROS, such as hydrogen peroxide (H_2_O_2_), play an important role in plant defense responses [24]. We next examined the effect of endogenous and exogenous ABA on the bacterial flagellin peptide flg22-induced H_2_O_2_ production. The results showed that 35S::*ABA2* had higher H_2_O_2_ levels under induction with 1 µM flg22 than wild-type, whereas *aba2-1* had the lowest H_2_O_2_ levels (Figure 2A). Interestingly, exogenous application of ABA reduced flg22-induced H_2_O_2_ production in wild-type plants when compared to water controls (Figure 2B). These results indicate that endogenous and exogenous ABA function differently during PAMP-induced ROS burst in *Arabidopsis*.

### 2.3. Exogenous ABA, but not Endogenous ABA, Enhanced Catalase Activity under PstDC3000 Infection in Arabidopsis

In *Arabidopsis*, the nicotinamide adenine dinucleotide phosphate (NADPH) oxidases, such as RBOHD and RBOHF, are mostly responsible for ROS generation during plant–pathogen interactions [25]. We examined whether endogenous and exogenous ABA differently regulate *RBOHD* and *RBOHF* transcripts in *Arabidopsis* under *Pst*DC3000 infection. Compared to wild-type, 35S::*ABA2* plants displayed an increase in the transcript levels of *RBOHD* and *RBOHF* (Figure S1). In contrast, *aba2-1* showed a reduced level of expression of these two genes under *Pst*DC3000 infection (Figure S1). ABA treatment also enhanced the expression of *RBOHD* and *RBOHF*, compared to water treatment (Figure S2). These results indicate that both endogenous and exogenous ABA upregulate *RBOHD* and *RBOHF* expression in *Arabidopsis* under *Pst*DC3000 infection.

**Figure 3:**
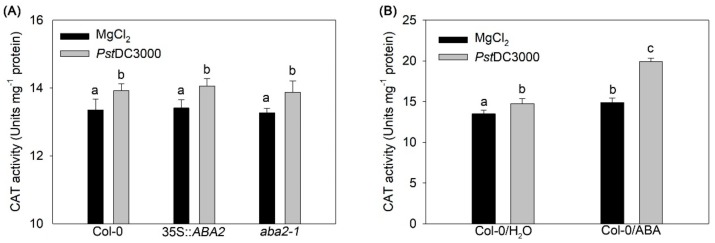
Effects of endogenous and exogenous abscisic acid (ABA) on catalase (CAT) activity under *Pst*DC3000 infection in *Arabidopsis* (**A**) Endogenous ABA and (**B**) exogenous ABA. For analyzing exogenous ABA, wild-type (Col-0) plants were treated with distilled water (H_2_O) or ABA before inoculation with *Pst*DC3000. Error bars indicate the standard error, and values were based on at least three independent replicates. Different letters indicate values that are significantly different (*p* < 0.05) from each other as determined by one-way ANOVA. These experiments were performed at least three times.

The alleviation of ROS-induced oxidative damage is often balanced via an efficient antioxidant defense system; for example, catalase (CAT) reacts with H_2_O_2_ to form oxygen and water [26]. We further detected whether CAT is differentially regulated by endogenous and exogenous ABA under *Pst*DC3000 infection. The results showed that CAT activity was not different between wild-type, 35S::*ABA2*, and *aba2-1* plants (Figure 3A). However, ABA treatment significantly enhanced CAT activity in wild-type plants under *Pst*DC3000 infection (Figure 3B).

### 2.4. RBOHD and RBOHF are Required for Endogenous ABA to Antagonize Exogenous ABA-Enhanced Bacterial Susceptibility in Arabidopsis

**Figure 4:**
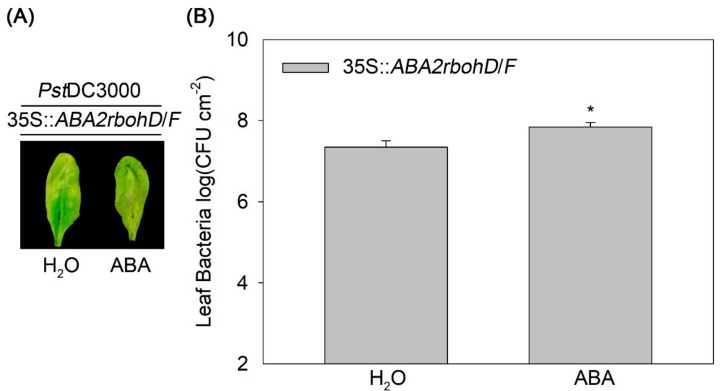
Effects of RBOHD and RBOHF on *Pst*DC3000 resistance in the *ABA2* transgenic *Arabidopsis* line 35S::*ABA2* at 3 dpi. (**A**) Disease symptoms and (**B**) bacterial growth. 35S::*ABA2rbohD*/*F* plants were treated with distilled water (H_2_O) or abscisic acid (ABA) prior to inoculation with *Pst*DC3000. Error bars indicate the standard error, and values were based on eight independent replicates. Asterisk indicates a significant difference (*t*-test, *p* < 0.05). These experiments were performed at least three times.

To investigate the mechanism of antagonism of 35S::*ABA2* plants to exogenous ABA-promoted *Pst*DC3000 susceptibility, we generated 35S::*ABA2rbohD*/*F* plants by crossing 35S::*ABA2* and the *rbohD*/*F* double mutant. The 35S::*ABA2rbohD*/*F* plants were pre-treated with ABA prior to inoculation with *Pst*DC3000. The results showed that ABA treatment enhanced disease symptoms and bacterial numbers in 35S::*ABA2rbohD*/*F* (Figure 4), indicating that *RBOHD* and *RBOHF* are required for endogenous ABA to antagonize exogenous ABA-aggravated bacterial susceptibility.

### 2.5. Both Endogenous and Exogenous ABA Repress Callose Formation and SA Signaling

**Figure 5:**
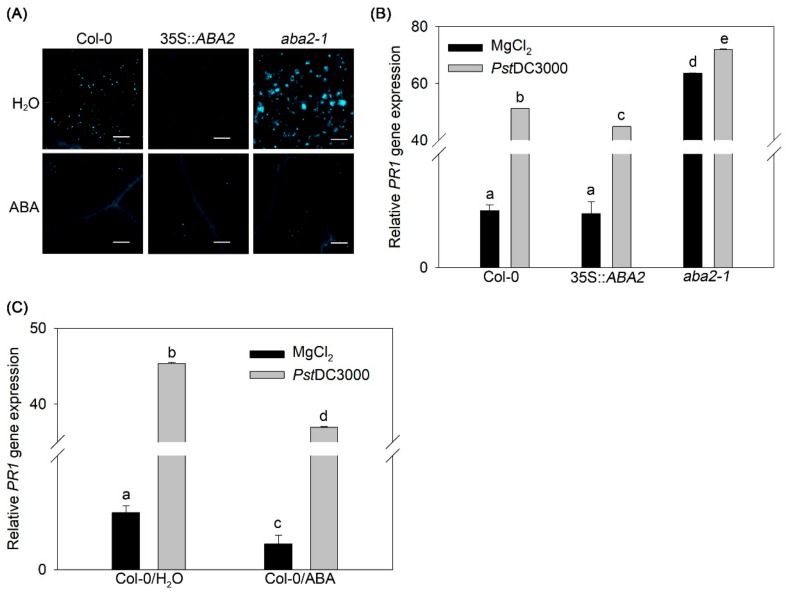
Effects of endogenous and exogenous abscisic acid (ABA) on callose formation and SA-dependent defense signaling in *Arabidopsis*. (**A**) Callose deposition and (**B**) and (**C**) *PR1* expression. Wild-type (Col-0), the *ABA2* transgenic line 35S::*ABA2*, and the *ABA2* gene mutant *aba2-1* were treated with distilled water (H_2_O) or ABA before inoculation with flg22 for detecting callose formation, and before inoculation with *Pst*DC3000 for detecting the expression of SA-dependent marker gene *PR1*. Scale Bars = 200 µm. Blue fluorescence means enhanced callose accumulation stained with Aniline blue. Error bars indicate the standard error, and values were based on at least three independent replicates. Different letters indicate values that are significantly different (*p* < 0.05) from each other as determined by one-way ANOVA. These experiments were performed at least three times.

In *Arabidopsis*, PAMP-induced callose formation has been widely used as a marker to characterize plant defense responses [27,28]. We assessed the effects of endogenous and exogenous ABA on flg22-induced callose generation by using wild type, 35S::*ABA2*, and *aba2-1* plants. With water treatment, 35S::*ABA2* showed no flg22-induced callose deposition, whereas *aba2-1* exhibited enhanced callose deposition compared to wild type (Figure 5A). Under ABA treatment, all tested plants showed no flg22-induced callose deposition (Figure 5A). These results suggest that both endogenous and exogenous ABA have similar functions in PAMP-induced callose deposition.

SA-dependent defense signaling has also been used as a marker to characterize plant defense resistance [29]. We next investigated the effect of endogenous and exogenous ABA on SA defense signaling during *Pst*DC3000 infection. qRT-PCR analysis showed that 35S::*ABA2* had lower SA-dependent *PR1* gene expression than wild-type plants, whereas *aba2-1* had higher *PR1* expression than wild type (Figure 5B). In addition, following *Pst*DC3000 infection, ABA treatment reduced *PR1* transcripts in wild-type plants, compared to water treatment (Figure 5C). These results indicate that endogenous and exogenous ABA function similarly in SA defense signaling during pathogen infection.

## 3. Discussion

Our data demonstrated that the *ABA2* overexpressing transgenic line 35S::*ABA2* showed no apparent response to exogenous ABA on *Pst*DC3000 multiplication (Figure 1). This indicates that endogenous ABA overlaps with exogenous ABA in regulating bacterial resistance. However, it is also possible that endogenous ABA antagonize exogenous ABA-promoted bacterial susceptibility. Interestingly, we found that endogenous and exogenous ABA function in opposing ways during flg22-induced H_2_O_2_ production (Figure 2). Analysis of gene expression demonstrated that both endogenous and exogenous ABA increased *RBOHD* and *RBOHF* transcripts (Figures S1 and S2). In addition, exogenous, but not endogenous ABA, enhanced CAT activity (Figure 3), thus giving an explanation for the difference between endogenous and exogenous ABA action on flg22-induced ROS burst. Loss of *RBOHD* and *RBOHF* restored the susceptibility of 35S::*ABA2* to exogenous ABA-promoted *Pst*DC3000 infection (Figure 4). These findings reveal that differential regulation of ROS production by endogenous and exogenous ABA is a main reason for the antagonistic effect of endogenous ABA on exogenous ABA-enhanced *Pst*DC3000 susceptibility in *Arabidopsis*.

Among ten NADPH oxidases in *Arabidopsis*, RBOHD and RBOHF play a main role in ROS generation during plant–pathogen interactions. Under recognition of PAMPs by the plasma membrane-localized receptor kinases (RKs) in plants, rapid cellular responses, such a burst of ROS are activated [25]. One of the best-studied RK signaling pathways is that the recognition of flg22 by FLAGELLIN-SENSING-2 (FLS2) [30] recruits BRI1-ASSOCIATED RECEPTOR KINASE 1 (BAK1), another RK as a co-receptor [31], to trans-phosphorylation of BOTRYTIS-INDUCED KINASE1 (BIK1), a cytoplasmic protein [32]. Phosphorylation of RBOHD by BIK1 is required for RBOHD activation [31]. In parallel to PAMPs-induced ROS burst, ABA also regulates ROS generation through RBOHD and RBOHF 24]. OPEN STOMATA 1 (OST1), one of ABA receptors, is required for ABA-induced ROS generation [33]. In addition, phosphorylation of OST1 by BAK1 is required for ABA-induced ROS generation in guard cells [34]. These findings indicate a crosstalk between PAMP and ABA-induced ROS production and imply that activation of RK signaling contributes to ABA-induced ROS generation. Since 35S::*ABA2* and *aba2-1* separately enhanced and reduced flg22-induced H_2_O_2_ production (Figure 2A), it is apparent that endogenous ABA contributes to PAMPs-induced ROS burst, and this contribution might be resulted from an underdetermined mechanism regulated by endogenous ABA. Despite ROS generation is required for both PAMPs- and ABA-induced stomatal immunity, but BIK1 is only required for stomatal closure induced by flg22 [24], indicating the difference between PAMP- and ABA-induced ROS generation. Our results that ABA treatment reduced flg22-induced H_2_O_2_ generation (Figure 2B) further reveal that exogenous ABA antagonizes PAMP-induced ROS production.

Indeed, it is not necessarily surprising for the different role of endogenous and exogenous ABA on ROS burst. On the one hand, ABA-responsive signal transduction is often involved in accumulation of H_2_O_2_ under abiotic stress [35]. It is likely that flg22 functions as abiotic stimulus. On the other hand, ABA limits H_2_O_2_ generation in some physiological processes, such as seed germination [36], and the transient alteration of ABA levels in the systemic leaf triggers acclimation of the systemic tissue [37]. It is possible that exogenous application of ABA prioritizes physiological processes by repressing flg22-induced H_2_O_2_ production.

ABA pre-treatment completely suppresses flg22-induced callose deposition in *Arabidopsis* [38]. ABA-hypersensitive lines and ABA treatment destroyed, whereas ABA-insensitive lines augmented, *Pst*DC3000-induced callose formation [39]. Our data that 35S::*ABA2* and *aba2-1* separately reduced and increased flg22-induced callose deposition, respectively (Figure 5A). Meanwhile, ABA treatment repressed flg22-induced callose deposition in wild-type plants (Figure 5A). These findings suggest that endogenous and exogenous ABA function as repressors in PAMP-induced callose deposition. A recent report showed that the expression of SA-responsive genes is higher in the ABA-deficient mutant *aba3-1* than in wild type, whereas ABA treatment restores the expression of SA-responsive genes in *aba3-1* to the levels seen in wild-type plants [21]. Our data that *aba2-1* increased, but 35S::*ABA2* and wild-type plants treated with ABA reduced the expression of SA-dependent *PR1* gene expression under *Pst*DC3000 infection (Figure 5B,C), indicate the similarity between endogenous and exogenous ABA in SA signaling, and support the antagonism of ABA in SA signaling in *Arabidopsis*. Considering the antagonism of endogenous ABA to exogenous ABA in disease susceptibility, our data further reveal that callose and SA are not related to this antagonistic relationship in *Pst*DC3000 susceptibility in *Arabidopsis*. Repression of callose deposition and SA signaling by both endogenous and exogenous ABA explains why 35S::*ABA2* and ABA-treated wild-type plants and *aba2-1* are all susceptible to *Pst*DC3000.

In conclusion, *aba2-1* and *35S::ABA2* characterized conversely on PAMP-induced ROS burst and callose deposition, as well as SA signaling, which; thus, removes the supposed effect of dwarfish of *aba2-1* on endogenous ABA function in plant defense responses. Particularly, we found that endogenous and exogenous ABA had completely converse function on PAMP-induced ROS burst, which is probably derived from differential regulation of ROS levels by endogenous and exogenous ABA affecting CAT activity. Since some pathogens are able to synthesize ABA and mediate endogenous ABA levels *in planta* [39,40,41], our results; thus, provide new insights into understanding the dual function of ABA in plants defending against bacterial pathogens.

## 4. Materials and Methods

### 4.1. Pathogens, Plants, and Growth Conditions

*Pst*DC3000 [42] was cultivated at 28 °C on King’s B (KB) medium (29 g Bacto^TM^ Proteose peptone, 1.5 g K_2_HPO_4_, 0.74 g MgSO_4_, 15 g Bacto^TM^ agar, and 8 g glycerol L^−1^) supplemented with Rifampicin (100 µg mL^−1^). *Arabidopsis thaliana* plants [43], including the *ABA2* transgenic line 35S::*ABA2* [23], *aba2-1* mutant [10], and *rbohD*/*F* double mutant [44], were all in the Columbia (Col-0) background. The transgenic line 35S::*ABA2* was crossed with the *rbohD*/*F* mutant. F2 homozygous individuals were identified by PCR using genotyping primers listed in Table S1. All plants were grown in a growth room at 23 °C, with 70% relative humidity, and 12/12 h photoperiod.

### 4.2. ABA Treatment and Bacterial Inoculation

For analyzing bacterial pathogenicity, ROS burst, callose deposition, and the expression of defense-related marker gene *PR1*, four-week-old *Arabidopsis* plants were sprayed with distilled water (H_2_O) as a control or 50 µM ABA. After 4 h, treated leaves were infiltrated with a suspension of 10^6^ colony forming units (cfu) mL^−1^ *Pst*DC3000 in 10 mM MgCl_2_. Photographs of leaves were taken at 3 dpi. These experiments were repeated at least three times.

### 4.3. Pathogenicity Analysis

After 3 days post inoculation (dpi) with virulent *Pst*DC3000, eight leaf discs (0.74 cm^2^ per disc, two leaf discs from one plant) per treatment were ground in 10 mM MgCl_2_ and used as one biological replicate. Dilutions of leaf homogenates with 10 mM MgCl_2_ were spotted onto KB agar medium containing 100 µg mL^−1^ Rifampicin. Eight biological replicates were harvested for each genotype used and each disease test was repeated at least three times.

### 4.4. Callose Deposition

Four-week-old *Arabidopsis* leaves were syringe-infiltrated with 1 µM flg22. After 24 h, inoculated leaves were collected and stained with Aniline blue [45].

### 4.5. Oxidative Burst

Four-week-old *Arabidopsis* leaves were sliced into strips of approximately 1 mm length and incubated overnight in distilled water in a 96-well plate. After removal of water from the 96-well plate, 1 µM flg22 in distilled water (H_2_O), supplemented with 20 mM luminol and 1 µg horseradish peroxidase (Sigma-Aldrich, Saint Louis, MO, USA), were added. Luminescence was recorded immediately with a GloMax^®^ 96 Microplate Luminometer (Promega, Madison, WI, USA).

### 4.6. Antioxidant Enzyme Activity

Total CAT activity of four-week-old *Arabidopsis* leaves was detected as previously reported [46].

### 4.7. qRT-PCR

Total RNA was extracted with RNeasy Plant Mini kit (Qiagen, Shanghai, China). RNA samples were treated with DNase Turbo DNAfree (Promega Madison, WI, USA). Total RNA (2 µg) was used for reverse transcription with SuperScript III reverse transcriptase (Invitrogen, Shanghai, China). qRT-PCR was performed using an Applied Biosystems 7500 Real-Time PCR System (Thermoscientific, Waltham, MA, USA) and SYBR Premix Ex Taq kit (TaKaRa, Otsu, Shiga, Japan). Gene transcripts were standardized using *ACT2* [47] as a control for *PR1*, *RBOHD*, and *RBOHF*. The primers are listed in Table S1.

## Supplementary Materials

Supplementary materials can be found at https://www.mdpi.com/1422-0067/20/10/2544/s1. Figure S1: Effects of endogenous ABA on *RBOHD* (A) and *RBOHF* (B) transcripts under *Pst*DC3000 infection in *Arabidopsis*. Error bars indicate the standard error, and values were based on at least three independent replicates. Different letters indicate values that are significantly different (*p* < 0.05) from each other as determined by one-way ANOVA. These experiments were performed at least three times. Figure S2: Effects of exogenous ABA on *RBOHD* (A) and *RBOHF* (B) transcripts under PstDC3000 infection in *Arabidopsis*. Wild-type (Col-0) plants were treated with distilled water (H2O) or ABA before inoculation with *Pst*DC3000. Error bars indicate the standard error, and values were based on at least three independent replicates. Different letters indicate values that are significantly different (*p* < 0.05) from each other as determined by one-way ANOVA. These experiments were performed at least three times. Table S1: List of genotyping primers.
