# Supplementary material for: Differential Function of Endogenous and Exogenous Abscisic Acid during Bacterial Pattern-Induced Production of Reactive Oxygen Species in Arabidopsis
Source: Int J Mol Sci. 2019 May 23;20(10):2544. doi: 10.3390/ijms20102544 (PMC6566928; doi:10.3390/ijms20102544)
Supplement: Supplementary file 1 [file ijms-20-02544-s001.pdf]

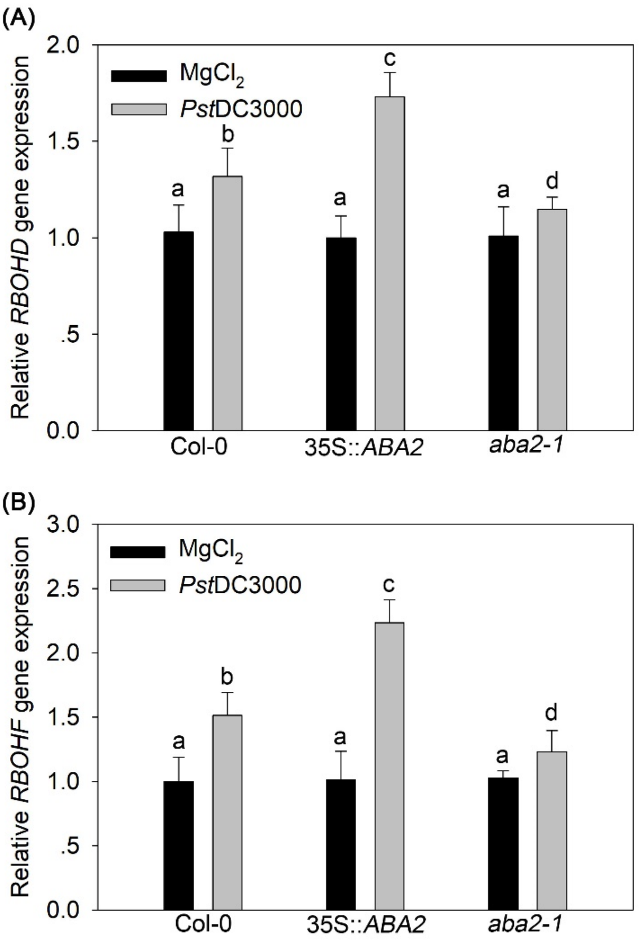

Figure S1. Effects of endogenous ABA on *RBOHD* (**A**) and *RBOHF* (**B**) transcripts under *Pst*DC3000 infection in *Arabidopsis*. Error bars indicate the standard error, and values were based on at least three independent replicates. Different letters indicate values that are significantly different ($p < 0.05$) from each other as determined by one-way ANOVA. These experiments were performed at least three times.

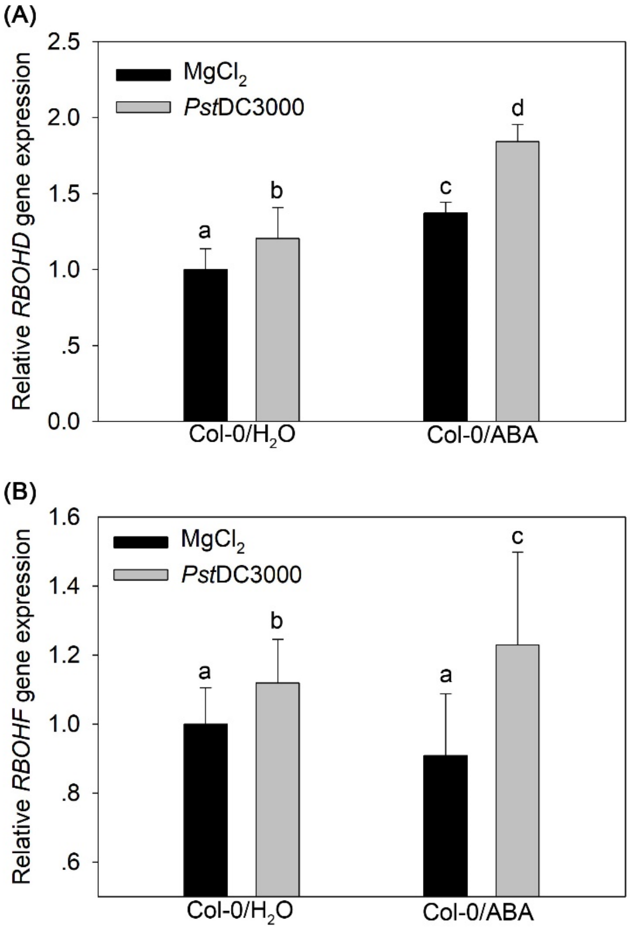

Figure S2. Effects of exogenous ABA on *RBOHD* (**A**) and *RBOHF* (**B**) transcripts under *Pst*DC3000 infection in *Arabidopsis*. Wild-type (Col-0) plants were treated with distilled water (H_2O) or ABA before inoculation with *Pst*DC3000. Error bars indicate the standard error, and values were based on at least three independent replicates. Different letters indicate values that are significantly different ($p < 0.05$) from each other as determined by one-way ANOVA. These experiments were performed at least three times.

Table S1. List of genotyping primers.

Mutant, Transgene, and RT-PCR	Primer Name: Sequence (5′→3′)
rbohD	rbohd-F: CCATTTAACGTGTGATTT
	rbohd-R: TCCATGTCGTTTTCTGAT
rbohF	rbohf-F: AACGATTCTCATGGTCTC
	rbohf-R: CTATGCATTGAGCGAAAT
35S::*ABA2*	35SABA2-F: CTCTGCCGACAGTGGTCC
	35SABA2-R: CACACCTCACCTCCGAGA
PR1	qpr1-F: TACTGGCTATTCTCGATT
	qpr1-R: CGTAAGGCCCACCAGAGT
RBOHD	qrbohd-F: ATTAGTCTTAGTGCTTCTGC
	qrbohd-R: GTCTAAGCTTCTGACTTAAC
RBOHF	qrbohF-F: GAGTGCATCTGCAAATAAGC
	qrbohF-R: CATTCTATGTATTCGACTCT
